# Deranged Liver Function Tests Associated With Hyperferritinemia in a Patient With Type 2 Diabetes Mellitus: A Case Study and Literature Review

**DOI:** 10.7759/cureus.66074

**Published:** 2024-08-03

**Authors:** Erwa Eltayib Elmakki

**Affiliations:** 1 Department of Internal Medicine, Faculty of Medicine, Jazan University, Jazan, SAU

**Keywords:** metabolic dysfunction-associated steatotic liver disease, metabolic liver disease, liver function tests, diabetes mellitus, hyperferritinemia

## Abstract

Metabolic dysfunction-associated steatotic liver disease (MASLD) is a common complication in patients with type 2 diabetes mellitus (T2DM), potentially progressing to more severe conditions such as metabolic dysfunction-associated steatohepatitis (MASH), liver cirrhosis, and hepatocellular carcinoma. This case study presents a 55-year-old man with long-standing T2DM who was found to have deranged liver function tests, elevated serum iron, and hyperferritinemia during a routine follow-up visit. The patient's clinical presentation, laboratory findings, and imaging results led to a diagnosis of MASH, complicated by dysregulated iron metabolism. This case highlights the importance of vigilant monitoring of liver function and iron studies in T2DM patients. Furthermore, it illustrates the challenges in managing the complex interplay between metabolic syndrome, liver dysfunction, and cardiovascular risk.

## Introduction

Diabetes mellitus (DM) is a chronic metabolic disorder characterized by elevated blood glucose levels resulting from the body's inability to produce or effectively use insulin [1]. Type 2 diabetes mellitus (T2DM) is the most common form, accounting for approximately 90% of all diabetes cases [1]. In 2023, three major international liver organizations proposed replacing the term "non-alcoholic fatty liver disease (NAFLD)" with "metabolic dysfunction-associated steatotic liver disease (MASLD)." Additionally, they suggested that the term "non-alcoholic steatohepatitis (NASH)" should be changed to "metabolic dysfunction-associated steatohepatitis (MASH) [2].

MASLD is a common liver condition associated with T2DM. In a recent study that was conducted in the United States, the prevalence of MASLD and MASH among individuals with T2DM was found to be 69.6% and 13.6%, respectively [3]. MASLD is characterized by the accumulation of fat in the liver, which can progress to more severe forms, such as MASH, fibrosis, and cirrhosis [4]. The development of MASLD in patients with T2DM is multifactorial, involving insulin resistance, obesity, and dysregulation of lipid metabolism [4]. Patients with MASLD often present with deranged liver function tests (LFTs), including elevated levels of liver enzymes, mainly alanine aminotransferase (ALT) and aspartate aminotransferase (AST) [5].

Abnormalities in serum iron studies are frequently observed in patients with MASH. Notably, elevated serum ferritin levels are a common finding, with studies indicating that hyperferritinemia is present in approximately 40-58% of MASH patients [5]. Emerging evidence suggests that dysregulated iron homeostasis, characterized by elevated serum ferritin levels, plays a significant role in MASLD pathogenesis. Furthermore, ferroptosis, a form of regulated cell death driven by iron-dependent lipid peroxidation, has been implicated in the progression of MASLD [6].

Deranged LFTs associated with hyperferritinemia can pose a diagnostic challenge, particularly in patients with comorbidities such as T2DM. Our goal in writing this case is to enhance understanding of best practices in diagnosing and managing MASH among diabetic patients, potentially improving patient outcomes in similar cases.

## Case presentation

A 55-year-old man known to have T2DM for 10 years was referred to our clinic because of deranged LFTs, mainly consisting of mild elevation of transaminases; otherwise, he was asymptomatic. Apart from T2DM, he had no other chronic condition nor previous history of blood transfusion or surgical intervention. Moreover, there was no family history of liver disease, and he denied alcohol consumption.

His medications included metformin 500 mg twice daily, and atorvastatin 10 mg once per day. General physical assessment revealed neither jaundice nor other peripheral stigmata of chronic liver disease. He had a blood pressure of 120/80, pulse of 84 beats/min, body mass index (BMI) of 32, and waist circumference of 100 cm. Physical examination of his chest abdomen and neurological systems was unremarkable. Initially, based on the clinical and laboratory data, we considered a list of differential diagnoses that are presented in Table 1. According to these differentials, a comprehensive work-up was performed and the results are deployed in Table 2.

**Table 1:** Differential diagnoses MASH: Metabolic dysfunction-associated steatohepatitis

Suggested Diagnoses
MASH
Hemochromatosis
Chronic viral hepatitis
Alcoholic hepatitis
Autoimmune hepatitis
Drug-induced hepatitis
Inherited liver diseases

**Table 2:** Summary of the performed studies Hb: Hemoglobin; MCV: Mean corpuscular volume; MCH: Mean corpuscular hemoglobin; MCHC: Mean corpuscular hemoglobin concentration; WBC: White blood cell count; ALT: Alanine aminotransferase; AST: Aspartate aminotransferase; ALP: Alkaline phosphatase; GGT: Gamma-glutamyl transferase; LDH: Lactate dehydrogenase; INR: International normalized ratio; FBG: Fasting blood glucose; HbA1c: Glycated hemoglobin; LDL-C: Low-density lipoprotein cholesterol; HDL: High-density lipoprotein cholesterol; HAV: Hepatitis A virus; HBsAg: Hepatitis B surface antigen; HCV: Hepatitis C virus; ANA: Antinuclear antibody; ASMA: Anti-smooth muscle antibody; TIBC: Total iron binding capacity

Investigations		
Test	Result	Normal values
Hb	14.6 g/dl	13.5-17.5 g/dL
MCV	82.2 fL	80-100 fL
MCH	27.4 pg	27-33 pg
MCHC	33.4 g/dL	32-36 g/dL
Reticulocytes	1.2%	0.5%-1.5%
WBC	7500 cells/μL	4,500-11,000 cells/μL
Platelets	178,000 cells/μL	150,000-450,000/μL
Total bilirubin	1.02 mg/dL	0.3-1.2 mg/dL
Unconjugated bilirubin	1.00 mg/dL	0.2-1.0 mg/dL
Total proteins	8.08 g/dL	6.0-8.3 g/dL
Albumin	4.94 g/dL	3.4-5.4 g/dL
ALT	120 U/L	10-40 U/L
AST	100 U/L	10-34 U/L
ALP	58.28 U/L	40-129 U/L
GGT	30 U/L	9-48 U/L
LDH	180 U/L	140-280 U/L
INR	1.2	<1.3
FBG	150 mg/dL	<100 mg/dL
HbA1c	8.00	<5.7%
Total cholesterol	220	<200 mg/dL
LDL-C	190	<100 mg
HDL	35	40-50 mg
HAV	Non-reactive	
Blood urea	20 mg/dL	
Serum creatinine	1 mg/dL	
HBsAg	Non-reactive	
HCV	Non-reactive	
ANA	Negative	
ASMA	Negative	
Serum iron	210 mcg/dL	60-170 mcg/dL
Serum ferritin	640 ng/mL	25-350 ng/mL
TIBC	430 mcg/dL	250-450 mcg/dL
Transferrin saturation	48.84%	20-50%
Genetic testing	No abnormal genes	
Liver ultrasound	Fatty liver	
Fibroscan	Grade 2 fibrosis (F2)	

Based on the patient's clinical data and the workup detailed in Table 2, the final diagnosis for this case was uncontrolled DM associated with metabolic syndrome, MASLD/MASH, and iron overload secondary to MASH. The patient was educated about his condition and advised to adopt lifestyle modifications, including weight loss, dietary changes, and increased physical activity. The patient's existing metformin therapy was continued at a dose of 750 mg twice daily. Semaglutide was added, starting at 3 mg orally daily for the first month, then increased to 7 mg daily thereafter. The dose of atorvastatin was increased to 20 mg daily. The patient was informed that regular monitoring of glycemic status, LFTs, iron profile, and assessment of liver fibrosis were crucial to monitor the progression of the disease and guide further management. However, unfortunately, the patient failed to attend the scheduled follow-up appointments.

## Discussion

The patient in this case has obvious features of metabolic syndrome including T2DM, obesity, dyslipidemia, and hypertension. Metabolic syndrome is a strong risk factor for MASH development [5]. Mild elevation of transaminases (ALT and AST) associated with iron overload in the setting of a long-standing T2DM are common features of MASH [5,6]. Given the patient's clinical scenario, MASH was the most likely diagnosis among the possible options that were being considered. Another important differential diagnosis in this case included hemochromatosis. However, the lack of other clinical features of hemochromatosis, normal transferrin saturation, and absence of a family history of liver disease as well as normal genetic studies, made this diagnosis unlikely. Additionally, the common etiologies of liver injury, such as viral hepatitis, alcoholic hepatitis, drug-induced and autoimmune hepatitis, and inherited metabolic liver diseases were appropriately considered and accordingly excluded.

Several studies have highlighted the complex relationship between hyperferritinemia, MASH, and T2DM. Elevated ferritin levels are frequently observed in patients with MASH, particularly those with concomitant T2DM. This association is thought to be due to a combination of factors, including insulin resistance-induced alterations in iron metabolism and increased oxidative stress. In addition, serum ferritin levels were independently associated with the severity of liver fibrosis in patients with MASH, suggesting that iron may play a role in disease progression [7,8].

Furthermore, a recent study reported that eight ferroptosis-related genes were elevated in patients with higher hepatic steatosis grade. This evidence indicates the essential role of iron metabolism and ferroptosis in the pathogenesis of MASLD/MASH [6].

A case report by Nelsen et al. presents notable similarities to our current case, offering valuable comparative insights [9]. Both cases exhibit hallmark features of metabolic syndrome, including poorly controlled T2DM, alongside deranged LFTs and hyperferritinemia. Interestingly, both patients maintained normal transferrin saturation levels, a finding that distinguishes these cases from classical hemochromatosis. However, key differences are observed in the severity of certain parameters. In our case, the patient presents with higher transaminase levels, potentially indicating a more pronounced hepatocellular injury. Conversely, the serum iron level in our patient is higher (220 mg/dl and 109 mg/dl, respectively) compared to the case reported by Nelson et al. [9]. These variations underscore the heterogeneity in the presentation of MASLD and highlight the need for individualized assessment and management strategies.

Imaging techniques are commonly used to detect fat infiltration in the liver. Liver ultrasound is generally sensitive for this purpose, while CT scans offer even higher sensitivity. However, these imaging methods cannot differentiate between MASLD and MASH [5]. Liver biopsy remains the gold standard for confirming MASH, but it is invasive and carries risks such as bleeding. To avoid these risks, non-invasive alternatives have been developed [5,9]. These include transient elastography (fibroscan), magnetic resonance elastography (MRE), and various non-invasive scores and biomarkers. These methods assess liver stiffness and fibrosis, helping to establish a diagnosis of MASH without the need for invasive procedures [4,5].

Management of MASH in patients with T2DM requires a multifaceted approach. Lifestyle modifications, including weight loss and increased physical activity, remain the cornerstone of treatment. A study reported that a 7-10% weight loss can lead to significant improvements in liver histology in MASH patients [10]. The latest (March 2024) clinical guidelines from the American College of Physicians (ACP) recommend adding a glucagon-like peptide-1 receptor agonist (GLP-1RA) medication to metformin for patients with T2DM who are not achieving adequate blood sugar control with metformin alone [11]. This approach can enhance diabetic control, reduce obesity, and lower the risk of cardiovascular events, stroke, and all-cause mortality [11]. The addition of a GLP-1RA medication, such as semaglutide or liraglutide, is especially relevant for the patient in this case. Glucagon-like peptide-1 (GLP-1) agonists have been shown to target not only T2DM but also the patient's comorbid conditions. Interestingly, GLP-1 agonists like liraglutide and semaglutide are significantly effective in treating MASLD/MASH. GLP-1 agonists can improve liver enzyme levels and reduce liver fat content in individuals with T2DM and MASH [12,13]. Furthermore, an even newer dual GLP-1 and gastric inhibitory polypeptide (GIP) agonist medication called tirzepatide has shown superior efficacy compared to placebo in achieving the resolution of MASH without worsening liver fibrosis, according to recently published trial data [10]. Pioglitazone, a thiazolidinedione class anti-diabetic medication has been recommended by the American Association for the Study of Liver Disease (AASLD) and the European Guidelines for treating diabetic patients with MASH [14]. This agent has shown promising results in not only halting but potentially reversing hepatic fibrosis. However, its use in MASH is considered off-label and the risk/benefit balance related to pioglitazone side-effects, particularly cardiac toxicity, should be discussed with each patient [14]. On the other hand, unlike pioglitazone, to date, the GLP-1 agonists do not have a significant effect on improving hepatic fibrosis [15].

Recently (in March 2024), the American FDA has granted accelerated approval to resmetirom (Rezdiffra) in adults for the treatment of non-cirrhotic MASH with moderate to advanced liver fibrosis (consistent with stages F2 to F3 fibrosis), to be used in conjunction with diet and exercise [16]. Resmetirom is an oral liver-directed, thyroid hormone receptor beta-selective agonist that has been shown to increase the metabolism of fat in the liver. This in turn decreases the buildup of harmful, toxic fat species in the liver and ultimately reduces the overall fat content within the liver [16].

It is crucial to emphasize that our studied case has a high risk for atherosclerotic cardiovascular disease (ASCVD). This risk is particularly significant given the well-established evidence that cardiovascular events, rather than hepatic complications, represent the primary cause of mortality among patients with MASH and metabolic syndrome [4,5]. Therefore, aggressive management of dyslipidemia is warranted. However, this presents a clinical dilemma: while lipid-lowering agents, particularly statins, are the cornerstone of ASCVD prevention, they carry a potential risk of hepatotoxicity [17,9]. Significantly, chronic liver disease and compensated cirrhosis are not considered contraindications to statin therapy. In contrast, decompensated cirrhosis and acute liver failure are contraindications to the use of statin medications [17]. Notably, recent evidence suggests that statins may be beneficial in MASH. A meta-analysis by Zhou et al. (2023) found that statin use in MASH patients was associated with improved liver enzymes and reduced hepatic steatosis [18]. Moreover, the cardiovascular benefits of statins in high-risk patients generally outweigh the low risk of clinically significant liver injury. Thus, a nuanced approach is necessary. Careful monitoring of LFTs is essential when initiating statin therapy in MASH patients. On the other hand, alternative lipid-lowering strategies, such as ezetimibe, a potent inhibitor of cholesterol absorption may be considered in patients with more advanced liver disease or those who cannot tolerate statins. Additionally, the decision to use statins should be individualized based on the patient's overall cardiovascular risk, liver function, and ability to tolerate the medication [19]. Recently, the iron-chelating agent deferoxamine has been proven to alleviate MASLD, including MASH, both in vitro and in vivo [6]. Furthermore, iron depletion therapy can be effective in reducing serum transaminase (ALT, AST) levels and improving insulin sensitivity in individuals with MASLD [8].

Notingly, the long-term prognosis for patients with MASH and diabetes is concerning as these conditions synergistically increase the risk of adverse outcomes. Patients with both MASH and T2DM had a significantly higher risk of developing hepatocellular carcinoma compared to those with either condition alone [20]. This underscores the importance of early detection and aggressive management of both conditions to mitigate long-term complications.

Finally, this case highlights the need for routine LFTs and iron studies in patients with long-standing T2DM, even in the absence of overt liver-related symptoms or signs. Future research should focus on elucidating the precise mechanisms linking iron metabolism, insulin resistance, and liver injury in MASH. Moreover, prospective studies are needed to evaluate the potential benefit of iron-lowering therapies in this patient population.

## Conclusions

The combination of deranged liver enzymes, elevated serum ferritin, in the setting of T2DM, and other elements of metabolic syndrome points to the development of MAFLD, which can potentially progress to MASH. The management of such cases requires a multifaceted approach, including a strict lifestyle approach, strict glycemic control, weight management, and addressing liver fibrosis and iron overload. Routine screening for liver disease among diabetic patients is essential, as early detection and intervention can help prevent the development of liver cirrhosis and hepatocellular cancer. This case emphasizes that healthcare providers should maintain a high index of suspicion for liver involvement in patients with long-standing T2DM, even in the absence of overt clinical signs. Prompt evaluation and a comprehensive management plan are crucial to mitigate the risk of advanced liver disease in this high-risk population.

## References

[1] American Diabetes Association (2021). Classification and diagnosis of diabetes: standards of medical care in diabetes - 2021. Diabetes Care.

[2] Targher G, Byrne CD, Tilg H (2024). MASLD: a systemic metabolic disorder with cardiovascular and malignant complications. Gut.

[3] Mittal N, Siddiqi H, Madamba E, Richards L, Bettencourt R, Ajmera V, Loomba R (2024). A prospective study on the prevalence of at-risk MASH in patients with type 2 diabetes mellitus in the United States. Aliment Pharmacol Ther.

[4] Chan WK, Chuah KH, Rajaram RB, Lim LL, Ratnasingam J, Vethakkan SR (2023). Metabolic dysfunction-associated steatotic liver disease (MASLD): a state-of-the-art review. J Obes Metab Syndr.

[5] Stengel JZ, Harrison SA (2006). Nonalcoholic steatohepatitis: clinical presentation, diagnosis, and treatment. Gastroenterol Hepatol (N Y).

[6] Shen X, Yu Z, Wei C, Hu C, Chen J (2024). Iron metabolism and ferroptosis in nonalcoholic fatty liver disease: what is our next step?. Am J Physiol Endocrinol Metab.

[7] Britton L, Bridle K, Reiling J (2018). Hepatic iron concentration correlates with insulin sensitivity in nonalcoholic fatty liver disease. Hepatol Commun.

[8] Nelson JE, Klintworth H, Kowdley KV (2012). Iron metabolism in nonalcoholic fatty liver disease. Curr Gastroenterol Rep.

[9] Nelsen EM, Newman DB, Sweetser S (2012). 52-year-old man with liver enzyme abnormalities and elevated ferritin level. Mayo Clin Proc.

[10] Loomba R, Hartman ML, Lawitz EJ (2024). Tirzepatide for metabolic dysfunction-associated steatohepatitis with liver fibrosis. N Engl J Med.

[11] Qaseem A, Obley AJ, Shamliyan T (2024). Newer pharmacologic treatments in adults with type 2 diabetes: a clinical guideline from the American College of Physicians. Ann Intern Med.

[12] Newsome PN, Buchholtz K, Cusi K (2021). A placebo-controlled trial of subcutaneous semaglutide in nonalcoholic steatohepatitis. N Engl J Med.

[13] Nevola R, Epifani R, Imbriani S (2023). GLP-1 receptor agonists in non-alcoholic fatty liver disease: current evidence and future perspectives. Int J Mol Sci.

[14] Associazione Italiana per lo Studio del Fegato (AISF), Società Italiana di Diabetologia (SID) and Società Italiana dell’Obesità (SIO) (2022). Non-alcoholic fatty liver disease in adults 2021: a clinical practice guideline of the Italian Association for the Study of the Liver (AISF), the Italian Society of Diabetology (SID) and the Italian Society of Obesity (SIO). Eat Weight Disord.

[15] Armstrong MJ, Gaunt P, Aithal GP (2016). Liraglutide safety and efficacy in patients with non-alcoholic steatohepatitis (LEAN): a multicentre, double-blind, randomised, placebo-controlled phase 2 study. Lancet.

[16] Mironova M, Sherker AH (2024). In NASH with liver fibrosis, resmetirom improved NASH resolution and reduced fibrosis at 1 y. Ann Intern Med.

[17] Cohen DE, Anania FA, Chalasani N (2006). An assessment of statin safety by hepatologists. Am J Cardiol.

[18] Zhou H, Toshiyoshi M, Zhao W, Zhao Y, Zhao Y (2023). Statins on nonalcoholic fatty liver disease: a systematic review and meta-analysis of 14 RCTs. Medicine (Baltimore).

[19] Nakade Y, Murotani K, Inoue T (2017). Ezetimibe for the treatment of non-alcoholic fatty liver disease: a meta-analysis. Hepatol Res.

[20] Otero Sanchez L, Chen Y, Lassailly G, Qi X (2024). Exploring the links between types 2 diabetes and liver-related complications: a comprehensive review. United European Gastroenterol J.

